# Editorial: Immunomodulatory molecules of natural origin: innovative strategies for combatting cancer

**DOI:** 10.3389/fonc.2025.1568568

**Published:** 2025-03-24

**Authors:** Lekshmi R. Nath, Gautam Sethi, Vijayasteltar B. Liju

**Affiliations:** ^1^ Department of Pharmacognosy, Amrita School of Pharmacy, Amrita Vishwa Vidyapeetham, Kochi, Kerala, India; ^2^ Department of Pharmacology, Yong Loo Lin School of Medicine, National University of Singapore, Singapore, Singapore; ^3^ The Shraga Segal Department of Microbiology, Immunology and Genetics, Faculty of Health Sciences, Ben-Gurion University of the Negev, Beersheva, Israel

**Keywords:** immunomodulation, cancer, natural compounds, immunotherapy, preclinical studies, phytomolecules

Cancer remains a major global health challenge, reinforcing the need for innovative therapeutic strategies to achieve better outcomes. Despite existing traditional therapies such as *radiation therapy*, *chemotherapy*, and *photodynamic therapy*, cancer mortality rates continue to rise each year. Recently, immunotherapy has gained significant attention for its role in reactivating antitumour immune responses to combat and control cancer. Based on target specificity, immunotherapy strategies are divided into two categories: *specific* and *nonspecific*. Specific immunomodulators, such as vaccines, are designed to target a specific antigen. In contrast, nonspecific immunomodulators enhance the anticancer immune response by activating both the innate and adaptive immune systems and by modulating immune cells such as *macrophages*, *dendritic cells*, and *T lymphocytes*, and also the tumour microenvironment (TME). Immune checkpoint inhibitors, such as antibodies targeting Programmed Death-1 (PD-1)/Programmed Death-Ligand 1 (PD-L1), and Chimeric Antigen Receptor T (CAR-T) cell therapy, have markedly enhanced treatment outcomes (1). However, despite these advancements, many patients remain unresponsive or only partially respond to treatments, and some develop immune-related adverse events (irAEs). This underscores the urgent need to develop new immunomodulators with enhanced safety profiles.

Phytomolecules currently being tested in clinical trials, such as *Apigenin*, *Berberine*, *Epigallocatechin gallate* (EGCG), *Curcumin*, *Resveratrol*, and *Ginsenoside Rh2*, have shown the potential in preclinical studies to trigger an immune response against cancer via the PD-1/PD-L1 pathway (2). *Apigenin* can promote the proliferation of T lymphocytes and induce T-cell-mediated cell death in breast cancer and melanoma cells. It can also increase CD4+ and CD8+ T-cell infiltration in melanoma (B16-F10)-bearing mice, stimulating T-cell immunity by decreasing PD-L1 expression (3, 4). Myeloid-derived suppressor cells (MDSCs) and Treg cells are potent immunosuppressive cells that facilitate immune evasion in the immunosuppressive tumour microenvironment (TME) by suppressing T-cell activity. *Berberine* can reduce the number of activated MDSCs and Treg cells, thus switching the TME from immunosuppression to immune activation (5). *Curcumin* can overcome immunosuppression by decreasing MDSC and Treg cell populations while increasing CD8+ T lymphocytes in the TME, activating T-cell immunity and improving the tumour-induced immunosuppressive environment in tongue carcinoma (6). *EGCG* prevented PD-L1 upregulation induced by epidermal growth factor (EGF) through the AKT pathway in Non-Small Cell Lung Cancer (NSCLC) cells (7). *Ginsenoside Rk1* can effectively reduce PD-L1 expression in NSCLC by inhibiting the NF-κB pathway. Additionally, *Ginsenoside Rh2* increased CD4+ and CD8+ T-cell infiltration in the tumour tissues of melanoma (B16-F10)-bearing mice (8). Resveratrol has the ability to stimulate the growth of γδ T lymphocytes and Treg cells in cultured peripheral blood mononuclear cells from healthy individuals (9). This Research Topic underscores the considerable immunoregulatory potential of natural agents and their prospective applications in clinical contexts, particularly in combination with immunotherapy for cancer treatment. It comprises two review articles and two research articles, which collectively highlight the critical importance of ongoing research in this domain. This Research Topic encourages further exploration into effectively integrating these natural compounds with immunotherapy, offering a promising avenue for more comprehensive and less toxic cancer treatments.

Park et al. reported that *Paulownin* can enhance NK cell activation and functional potency by increasing the expression of CD107a and Granzyme B via activation of the JNK signalling pathway. The study demonstrates that *Paulownin* directly increases the cytolytic activity of NK cells at lower doses against solid tumour cell lines. *Paulownin* reduced melanoma tumour growth in mice by approximately 60%, with the effect being dependent on NK cell activity. In a comprehensive review article, Meng et al. analysed recent research done on the antitumour effect of *Psoralen*, focusing on the molecular mechanisms of its hepatotoxicity, and highlighting the need for the development of antitumour drugs that are both highly effective and low in toxicity. Immunostimulatory cytokines and their agonists can enhance anti-tumour immunity, as shown by agents targeting transforming growth factor-β (TGF-β). TGF-β can activate both the canonical TGFβ-Smad signalling pathway and non-canonical pathways that interact with others such as PI3K-AKT, ERK, and NF-κB, all of which are pro-tumorigenic and immunosuppressive and are often upregulated in advanced cancers. Over-activation of TGF-β signalling in cancer cells can inhibit dendritic cell (DC) maturation, chemotaxis, and the expression of components essential for antigen presentation, while also promoting Treg cell activation and immune checkpoint expression (10).

Fathima et al. studied the anticancer potential of *Withametelin* (WM) on HCT-116 colon cancer cells. WM displayed notable cytotoxicity on cancer cells with an IC50 of 0.719 ± 0.12 mM with a limited effect on non-cancerous cells. WM can function as an inhibitor of TGF-β signalling by reducing SMAD2/3 phosphorylation. WM suppressed TGF-β induced metastatic dissemination of cells by reducing the expression of various EMT markers such as N-cadherin, Snail, and Slug. TGF-β also enhanced the immunosuppressive tumour milieu by enhancing the proliferation of Treg cells and immune checkpoints. WM inhibited the expression of PD-L1, an immune checkpoint molecule associated with tumour immune evasion. This study emphasised the potential of WM to target two important aspects of cancer management: cancer cell death and anti-tumour immunity.

Ni et al. discussed the possibility of integrating Chinese herbal medicine (CHM) into the allopathic system to enhance its efficacy. Multiple studies have shown that a combination of CHM with chemotherapy can improve the treatment sensitivity, reduce toxicity and confer strong anti-tumour immunity. Clinical trials of Chinese proprietary medicines have demonstrated improved immune function and reduced chemotherapy-related side effects. CHM can also target multidrug resistance signalling in cancer cells. The review suggests that a better understanding of the mechanisms of action and the conduct of detailed clinical studies with CHM could facilitate its integration into mainstream clinical practice for cancer prevention and treatment. Moreover, comprehensive clinical trials are essential to establish the efficacy, safety, and optimal usage of CHM, thereby providing a stronger evidence base for its use alongside conventional cancer therapies. This approach could not only broaden the therapeutic arsenal but also might offer potential synergies that could improve patient outcomes and reduce the side effects associated with conventional cancer therapies. Refining the mechanism of action and detailed clinical studies with CHM support its clinical application for cancer prevention and treatment.

In conclusion, immunotherapy fundamentally reshapes the immune system to target and eradicate tumour cells at their source and has thus been approved for managing multiple types of cancer. However, its clinical utility is hampered by toxic side effects, resistance to treatment, lack of reliable prognostic markers, and high cost. Plant-derived bioactives interact with different components of the immune system, enhancing its ability to identify and destroy cancer cells while preserving healthy tissues. These natural agents can serve as supplements to other cancer therapies, such as chemotherapy, radiotherapy, and photodynamic therapy, to enhance the immune response, increase the efficacy of treatments, and mitigate the development of resistance. However, additional work is needed to better understand their mechanisms of action and to assess their safety and effectiveness in clinical studies.
